# Circulating D-Dimers Increase the Risk of Mortality and Venous Thromboembolism in Patients With Lung Cancer: A Systematic Analysis Combined With External Validation

**DOI:** 10.3389/fmed.2022.853941

**Published:** 2022-03-02

**Authors:** Jing Li, Shanle Yan, Xiaohui Zhang, Mengqi Xiang, Chuanhua Zhang, Ling Gu, Xiaoying Wei, Chuanyun You, Shenhua Chen, Daxiong Zeng, Junhong Jiang

**Affiliations:** ^1^Department of Medicine, Respiratory, Emergency and Intensive Care Medicine, The Affiliated Dushu Lake Hospital of Soochow University, Suzhou, China; ^2^Department of Rheumatology and Immunology, The Affiliated Dushu Lake Hospital of Soochow University, Suzhou, China; ^3^Department of Medicine, Respiratory, Emergency and Intensive Care Medicine, The First Affiliated Hospital of Soochow University, Suzhou, China; ^4^Department of Medical Oncology, Sichuan Cancer Hospital, Medical School, University of Electronic Science and Technology of China, Chengdu, China; ^5^Department of Thoracic Surgery, Sichuan Cancer Hospital, Medical School, University of Electronic Science and Technology of China, Chengdu, China

**Keywords:** D-dimer, lung cancer, mortality, meta-analysis, VTE

## Abstract

**Background:**

D-dimer is a fibrin-degrading substance that is soluble and whose degradation is produced by plasma protein-mediated degradation of cross-linked fibrin. Previous investigations have shown a link between D-dimer and the mortality in lung cancer patients. However, different investigations varied whether D-dimer could predict prognosis in these patients.

**Methods:**

A meta-analysis and systematic review of all available cohort studies were performed on the link between circulating D-dimer levels and survival of lung cancer patients. Relevant studies were searched in Embase, Cochrane Library, and PubMed databases. Data from 540 lung cancer patients from the First Hospital of Soochow University and Sichuan Cancer Hospital were used for external validation.

**Results:**

We finally obtained 19 eligible cohort studies with pooled HR showing that high D-dimer levels contribute to death in tumor group (HR 1.62, 95% CI: 1.39–1.88, I^2^ = 75.0%). Further stratified analysis showed that higher circulating D-dimer in the advanced lung cancer group was linked to a 1.91-fold risk (HR = 2.91, 95% CI: 2.24–3.78, I^2^ = 6.0%). Incorporation of other variables, including days of follow-up, country, design, public year, population, disease status, and quality score, into the meta-regression model, indicated that disease status was an additional source of heterogeneity (*p* < 0.001). External validation of 540 patients also showed that high levels of D-dimer showed a higher risk of overall mortality (HR 1.39, 95% CI: 1.13–1.72, *p* = 0.002) and VTE events (HR 3.98, 95% CI: 1.99–8.70, *p* = 0.002) in lung cancer patients.

**Conclusions:**

High circulating plasma D-dimer levels independently predict long-term prognosis and the risk of venous thromboembolism in lung cancer.

## Introduction

Malignant tumors of the respiratory system are the most frequently occurring malignancies and the primary cause of mortality in patients over 65 years old (1). Statistically, 1.8 million people are diagnosed with NSCLC or SCLC, while nearly 1.6 million dies of this disease in the world per year (2). The 5-year overall survival of NSCLC is only 17.7%, which is much lower than other cancers (3). Complications of lung cancer, especially coagulation disorders, are needed to be focused on. Previous studies confirmed that coagulation disorders occurred in those patients Incidence of hypercoagulability in malignant tumors often increased, such as venous thrombosis (VTE), disseminated intravascular coagulation (DIC), compared with the patients without cancer (4). Cancer-associated thrombosis predicted poor clinical prognosis and was regarded as the 2nd cause of mortality among patients with tumors (5). Besides, the risk of arterial and venous thromboembolism is significantly increased in patients with tumors. Pulmonary embolism (PE) is one of the most severe complications in those patients, and it is also a significant risk factor of cancer-related mortality worldwide (6, 7).

D-dimer is a fibrin-degrading substance that is soluble and whose degradation is produced by plasma protein-mediated degradation of cross-linked fibrin. It is regarded as a marker of fibrinolysis and coagulation (8). As a sign of hypercoagulability, D-dimer has been clinically used to evaluate the risk of VTE, DVT, and PE (9–11). D-dimer levels raised in patients with hypercoagulability and elevated while inflammation, malignancy, sepsis, recent surgery, trauma, severe burns, chronic kidney failure, and pregnancy (10). Previous research showed that elevated D-dimer levels predict poor prognosis in most types of tumors, such as lung, colorectal, pancreatic, or gastric cancer (4, 5, 12, 13).

However, the link between D-dimer and OS of these patients is still controversial. Several studies have shown that lung cancer cases with elevated levels of D-dimer had a poor prognosis (4, 14, 15). Some other research suggested no clear relationship between D-dimer levels and the prognosis statistically (16). Some studies show that patients with elevated D-dimer have a better prognosis (17, 18). Our ultimate purpose is to figure out the predictive value of D-dimer in these patients and provide better guidance for clinical intervention. We launched a meta-analysis to assess the value of D-dimer levels in patients with lung carcinomas. Data from multi-centers were used for external validation.

## Methods

### Patients

We accordingly conducted a cohort study to collect data including 540 NSCLC patients at our medical center and Sichuan Cancer Hospital between February 1, 2014, and March 1, 2016. The deadline for follow-up was December 1, 2021. The diagnosis of lung cancer was regarding pathological diagnostic criteria. Death data were obtained from the hospital registry, and the time of death was confirmed by telephone follow-up or by reviewing electronic medical records. The median follow-up period was 22.3 months. Informed consent was got from the patients themselves or their immediate family members. All research projects conformed to the guidelines of the ethics committees of Soochow University and Sichuan Cancer Hospital and followed the Declaration of Helsinki.

### Search Method

Databases from the Cochrane Library Embase and PubMed were categorized and searched from inception to December 31, 2021. The methods used followed the Meta-Analysis of Observational Studies in Epidemiology (MOOSE) and PRISMA guidelines for systematic analysis reporting statements (19). Supplementary Material shows data extraction, selection criteria, PRISMA checklist, search terms, and quality evaluation.

### Selection Strategy

The inclusion criteria for this meta-analysis and systematic review were as follows. (1) the subjects were human; (2) the data contained plasma or serum D-dimer levels; (3) death was shown as the primary endpoint and VTE events as a secondary endpoint; and (4) confidence intervals (CI) and hazard ratios (HRs) were obtained between D-dimer levels and patient death. We excluded case reports, animal studies, studies that provided insufficient data, or narrative reviews. Three independent reviewers performed literature reviews and database searches. A senior researcher consulted to resolve the above issues in case of disputes.

### Data Collection

For each potential inclusion, the first author's last country of origin, name, year of publication, gender, sample size, study design, age, serum or plasma D-dimer, adjustment variables, follow-up period, disease outcome, and their corresponding 95% CI and disease status were extracted. The primary endpoint analyzed in the meta-analysis was patient death. Three reviewers also performed data extraction, and senior investigator input was sought when disputes were encountered.

### Quality Assessment

We tested the risk of bias of the selected projects according to the Newcastle-Ottawa scale (20), including identifying exposure or outcome and group comparability. The GRADE scoring system analyzed the quality of evidence from the included studies in Grade Pro 4.05, and the relevant results are shown in Supplementary Table 1. Three reviewers independently assessed this process.

### Statistical Analysis

We analyzed the correlation of different levels of plasma D-dimer with patient death endpoints by confidence intervals and HR. We referred to various literature's original definitions as the quartiles of baseline plasma D-dimer. Cochran's Q test, I^2^ statistic, and *P*-value were used to assess the overall heterogeneity of the included studies. The merging process used a random-effects model for groups with several studies less than or equal to 5 in the subgroup analysis. In contrast, both random-effects models and fixed effects were used to calculate pooled HR or OR when heterogeneity was zero. A fixed-effects model was used. We further investigate the issue of the source of heterogeneity through stratified analysis. Our study also analyzed meta-regression models consistent with standard variables (including grade score, design, country, sample, days of follow-up, public years, clinical adherence, population, and adjusted covariates) to analyze heterogeneity similarly. We examined the effect of individual studies on overall heterogeneity by sensitivity analysis. We similarly analyzed funnel plot asymmetries to analyze publication and selective reporting bias. We also performed linear regression analysis by Egger and Begg to determine the statistical significance of the included studies when combined (21). Fill and trim methods were used to identify the number of additional items needed to provide adjusted effects and overcome potential biases. The mean ± standard deviation was presented as customarily distributed data, and the median and the IQR were presented as skewed data. Unpaired *t*-tests and Mann-Whitney U tests were used for comparison. Categorical variables were tested using the κ^2^-test and shown as percentages. Survival analysis was presented by Kaplan-Meier curves and analyzed using the log-rank test. Cox regression models examined multivariate and univariate survival analyses of overall survival (OS). Forest plots demonstrated the significance of the multifactorial analysis variables on prognosis. All statistical analyses were performed with RStudio (R version 4.0.2). Bilateral *P* < 0.05 were regarded as statistically significant.

## Result

### Literature Selection

**Figure 1:**
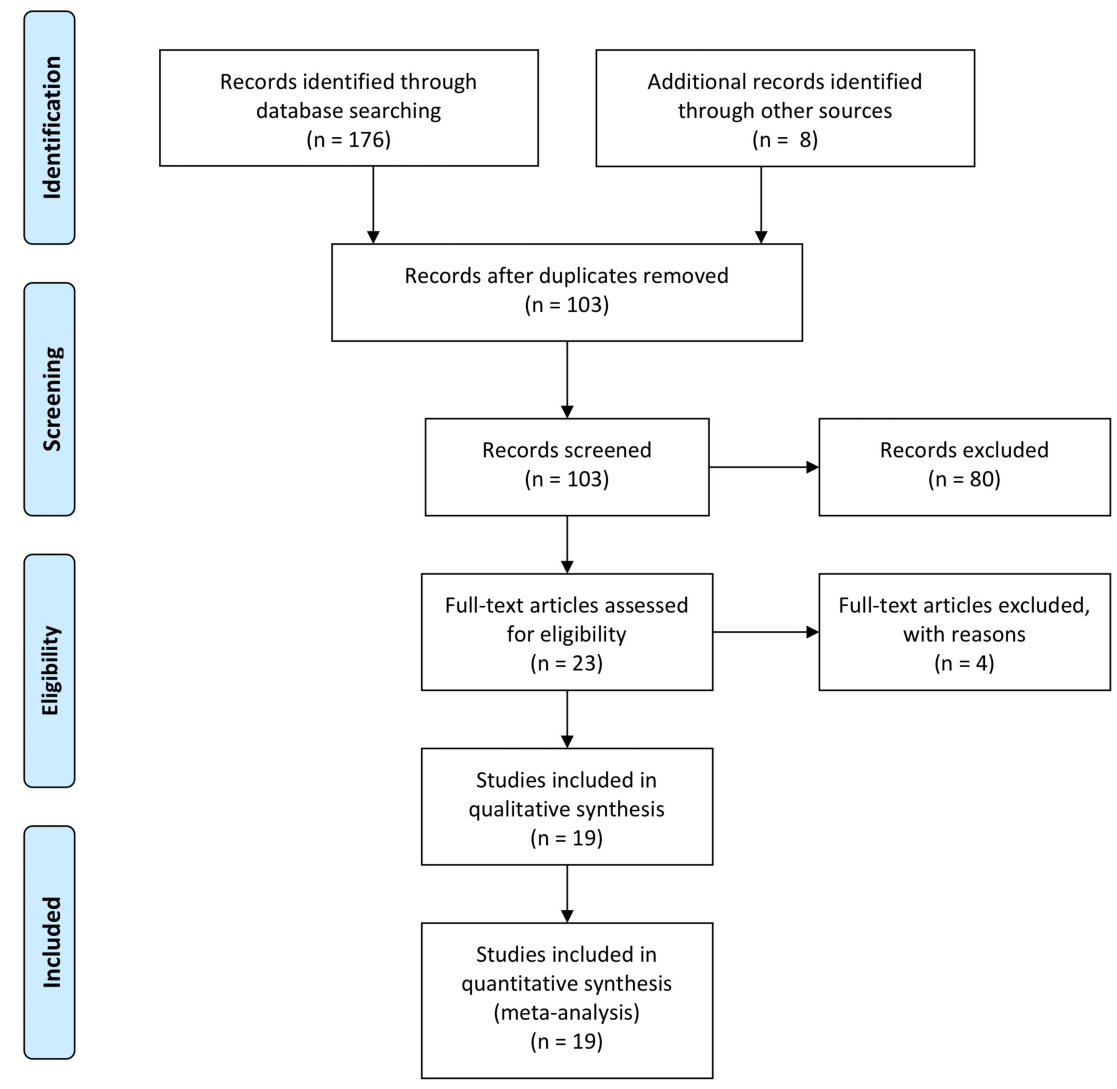
Flow chart of projects' selection designed by PRISMA.

We retrieved 176 records from Cochrane Library, Embase, and PubMed databases. Seventy-three of these records were duplicates, and for the remaining 103 records, we made further checks of the abstracts and titles. Of these records, 13 were not clinical studies, and 67 were unrelated to the subject. After the full-text screening of the final 23 records and removing the four studies that could not extract data (Three lacked data from D-dimer-related HRs, and one other primary source was children), we obtained 19 studies for final analysis. We show a flow chart of the projects' selection process in Figure 1.

### Study Baseline Data

The baseline information of the 19 projects published from 2007 to 2019 is displayed in Table 1. Eleven were launched in China (9, 14–18, 22–26), while others were performed in Poland (27, 28), Japan (4, 29, 30), Austria (31) and Turkey (13, 32), respectively. The follow-up time for these programs ranged from 11.5 to 60 months. Adjusted covariates of different studies are shown in Supplementary Table 2. Overall, the sample sizes of the project cohorts ranged from 52 to 1931, with a final sample size of 5,819 included in the meta-analysis. The study endpoint for all these projects was patient death. All included projects were ranked according to study quality, with projects scoring between 5 and 8 on the Newcastle-Ottawa Scale (NOS).

**Table 1:** Characteristics of included studies.

**Authors**	**Year**	**Country**	**Population**	**Stage**	**Follow-up months**	**Subjects**	**male(%)**	**Age**	**Assay**	**sample**	**Design**	**Quality score**
Fan, S.	2019	China	SCLC	NALC	60	82	81.7	60 (28–82)	IA	Plssma	RS	3
Minglei, Y.	2019	China	NSCLC	NALC	24	376	60.6	58 (27–81)	IA	Plssma	RCT	8
Hou, C.	2019	China	NSCLC	NALC	13.2	395	60.0	61 (35–81)	IA	Plssma	PCS	6
Huagang, L.	2019	China	NSCLC	NALC	42	456	69.7	65 (56–69)	IA	Plssma	RS	4
Edyta I. W.	2018	Poland	LC	ALC	30	95	75.0	67 (40–81)	IA	Plssma	PCS	6
Cuicui, Z.	2018	China	SCLC	NALC	60	160	80.6	59 (23–83)	IA	Plssma	RS	3
Wenwen, S.	2017	China	NSCLC	ALC	56	785	60.0	≥65 (41.6%)	IA	Plssma	PCS	5
Kaoru, S.	2017	Japan	NSCLC	ALC	60	237	59.1	69 ± 9.7	IA	Plssma	PCS	7
Zhu, L.-R.	2016	China	SCLC	NALC	60	74	77.0	57 (42–80)	IA	Plssma	PCS	6
Magdalena, Z.	2016	Poland	NSCLC	NALC	11.5	52	38.5	63 (58–70)	IA	Plssma	PCS	7
Koichi, F.	2015	Japan	NSCLC	ALC	60	247	61.5	69 (31–85)	IA	Plssma	PCS	6
Yuezhen, W.	2015	China	NSCLC	NALC	60	1931	70.0	≥65 (39.9%)	ELISA	Plssma	RS	5
Tuba, I.	2015	Turkey	LC	ALC	60	72	77.8	≥60 (37.5%)	IA	Plasma	PCS	6
Ge, L.-P.	2014	China	NSCLC	NALC	36	82	67.0	64 (44–72)	IA	Plasma	PCS	6
Heguo, J.	2014	China	NSCLC	ALC	36	184	45.2	≥60 (34.1%)	IA	Plasma	PCS	5
Zhang, P.-P.	2013	China	NSCLC	NALC	60	232	64.2	61 (30–86)	IA	Plasma	PCS	6
Cihan, A.	2012	Austria	LC	NALC	24	182	56.5	62 (52–68)	IA	Plasma	PCS	7
Katsuhiro, M.	2011	Japan	NSCLC	NALC	60	99	71.8	72 (35–88)	ELISA	Plasma	PCS	6
Altiay, G.	2007	Turkey	LC	ALC	78	78	93.6	61 (37–82)	ELISA	Plasma	PCS	7

*SCLC, small cell lung cancer; NSCLC, non-small-cell lung cancer; LC, lung cancer; NALC, non-advanced lung cancer; ALC, advanced lung cancer; IA, immunoturbidimetric assay; ELISA, enzyme-linked immunosorbent assay; RS, retrospecitive study; PCS, prospective cohort study; RCT, randomized controlled trial*.

### D-Dimer and Mortality

**Figure 2:**
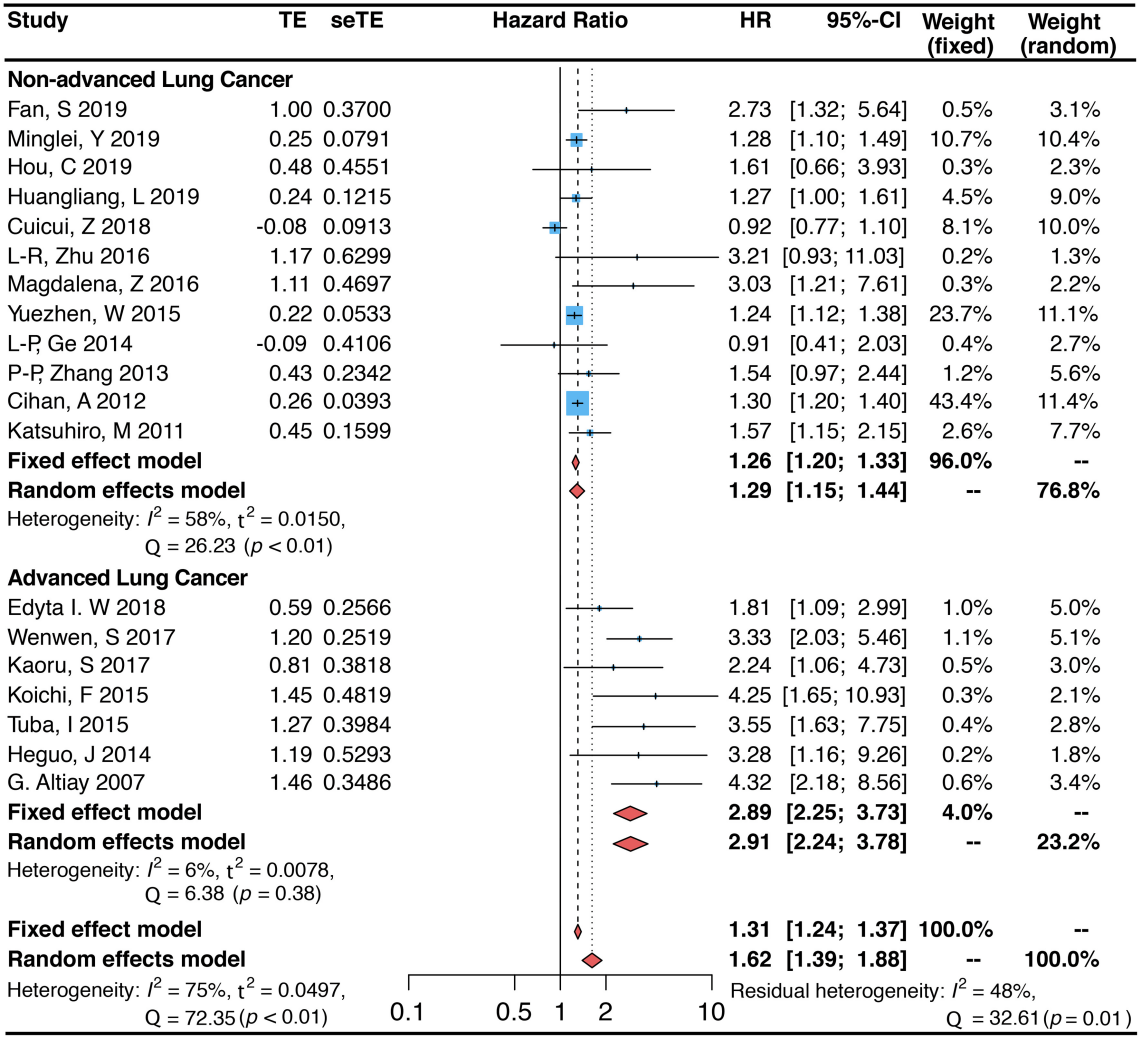
Forest plot (Fixed and random-effects model) for the link between plasma D-dimer levels (highest *vs*. lowest category) and lung cancer-associated mortality. Subgroup analysis is grouped by disease states (non-advanced lung cancer and advanced lung cancer).

The 19 projects we included, with an overall sample size of 5,819, were all studies that addressed the association between circulating blood D-dimer and the risk of death in patients with lung cancer (4, 9, 13–15, 22–32). Meta-analysis results from these 19 studies demonstrated that elevated circulating blood D-dimer levels were linked to an increased risk of death in patients with lung cancer (random-effects model. HR 1.62, 95% CI: 1.39–1.88; fixed-effects model. HR 1.31, 95% CI: 1.24–1.37). However, substantial heterogeneity remained between the different items included (I^2^ = 75.0%, *p* < 0.01; Figure 2). To analyze the sources of heterogeneity, we performed subgroup analyses to assess the effect of sample, country, design, population, public year, days of follow-up, disease status, and quality score on heterogeneity. The results showed that subgrouping by this classification resulted in the highest level of I^2^ decline in non-advanced lung cancer (HR = 1.29, 95% CI: 1.15–1.44, I^2^ = 58.0%) and advanced lung cancer (HR = 2.91, 95% CI: 2.24–3.78, I^2^ = 6.0%), indicating that this is likely the main source of heterogeneity (Figure 2). Studies of patients with either SCLC or NSCLC showed that high D-dimer levels increased the risk of death in patients with lung cancer (SCLC: HR = 1.86, 95% CI: 1.33–2.61, I^2^ = 85.0%; NSCLC: HR = 1.60, 95% CI: 1.33–1.92, I^2^ = 65.0%; Table 2). The number of studies in most subgroups was less than 5, so we used fixed-effects models to calculate pooled HRs (RS: HR = 1.21, 95% CI: 0.96–1.53; PCS: OR = 1.93, 95% CI: 1.57–2.37). In addition, the pooled HR (HR = 2.22, 95% CI: 1.58–3.13) was not statistically significant in the group from other countries than China, while the study from China showed a 44% risk of death in patients with increased D-dimer (HR = 1.44, 95% CI: 1.19–1.75). Analysis of other subgroups showed that the risk of death varied with the duration of follow-up (follow-up <60 months. HR = 1.52, 95% CI: 1.26–1.83, I^2^ = 64.0%; follow-up ≥60 months. HR = 1.89, 95% CI: 1.42–2.51, I^2^ = 82.0%). In addition, the association between plasma D-dimer and death persisted after inclusion in the new study, as can be shown by data from meta-regression (*P* = 0.794). The inclusion of other variables, including the year of disclosure, country, days of follow-up, population, quality score, disease status, and design in the meta-regression model, showed that disease status was likely the main source of heterogeneity (*P* < 0.001; Table 2). Data from sensitivity analyses showed that individual studies had little effect on overall heterogeneity (Supplementary Figure 1).

**Table 2:** Stratified analysis of pooled hazard ratio risks of D-dimer in LC patients.

**Stratified**	**Pooled HR**	**Heterogenety**	**Meta regression**
**analysis**	**(95% CI)**		**(*P*-value)**
Public year			0.794
After 2015	1.70 [1.38–2.10]	Q = 54.51, *P* < 0.01, I^2^ = 78.0%	
Before 2015	1.67 (1.22–2.29)	Q = 16.95, *P* < 0.01, I^2^ = 71.0%	
Country			0.121
China	1.44 [1.19–1.75]	Q = 38.22, *P* < 0.01, I^2^ = 74.0%	
Non-China	2.22 [1.58–3.13]	Q = 30.27, *P* < 0.01, I^2^ = 77.0%	
Population			0.683
SCLC	1.86 [1.33–2.61]	Q = 40.12, *P* < 0.01, I^2^ = 85.0%	
NSCLC	1.60 [1.33–1.92]	Q = 31.52, *P* < 0.01, I^2^ = 65.0%	
Follow-up months			0.519
≥60 months	1.89 [1.42–2.51]	Q = 49.03, *P* < 0.01, I^2^ = 82.0%	
<60 months	1.52 [1.26–1.83]	Q = 22.44, *P* < 0.01, I^2^ = 64.0%	
Design			0.074
RS	1.21 [0.96–1.53]	Q = 13.8, *P* < 0.01, I^2^ = 78.0%	
PCS	1.93 [1.57–2.37]	Q = 49.21, *P* < 0.01, I^2^ = 72.0%	
Quality Score			0.537
<6	1.55 [1.15–2.09]	Q = 33.88, *P* < 0.01, I^2^ = 85.0%	
≥6	1.74 [1.43–2.12]	Q = 34.18, *P* < 0.01, I^2^ = 65.0%	
Stage			<0.001
NALC	1.29 [1.15–1.44]	Q = 26.23, *P* < 0.01, I^2^ = 58.0%	
ALC	2.91 [2.24–3.78]	Q = 6.38, *P* = 0.38, I^2^ = 6.0%	

*SCLC, small cell lung cancer; NSCLC, non-small-cell lung cancer; LC, lung cancer; NALC, non-advanced lung cancer; ALC, advanced lung cancer; RS, retrospecitive study; PCS, prospective cohort study; HR, hazard ratio*.

**Figure 3:**
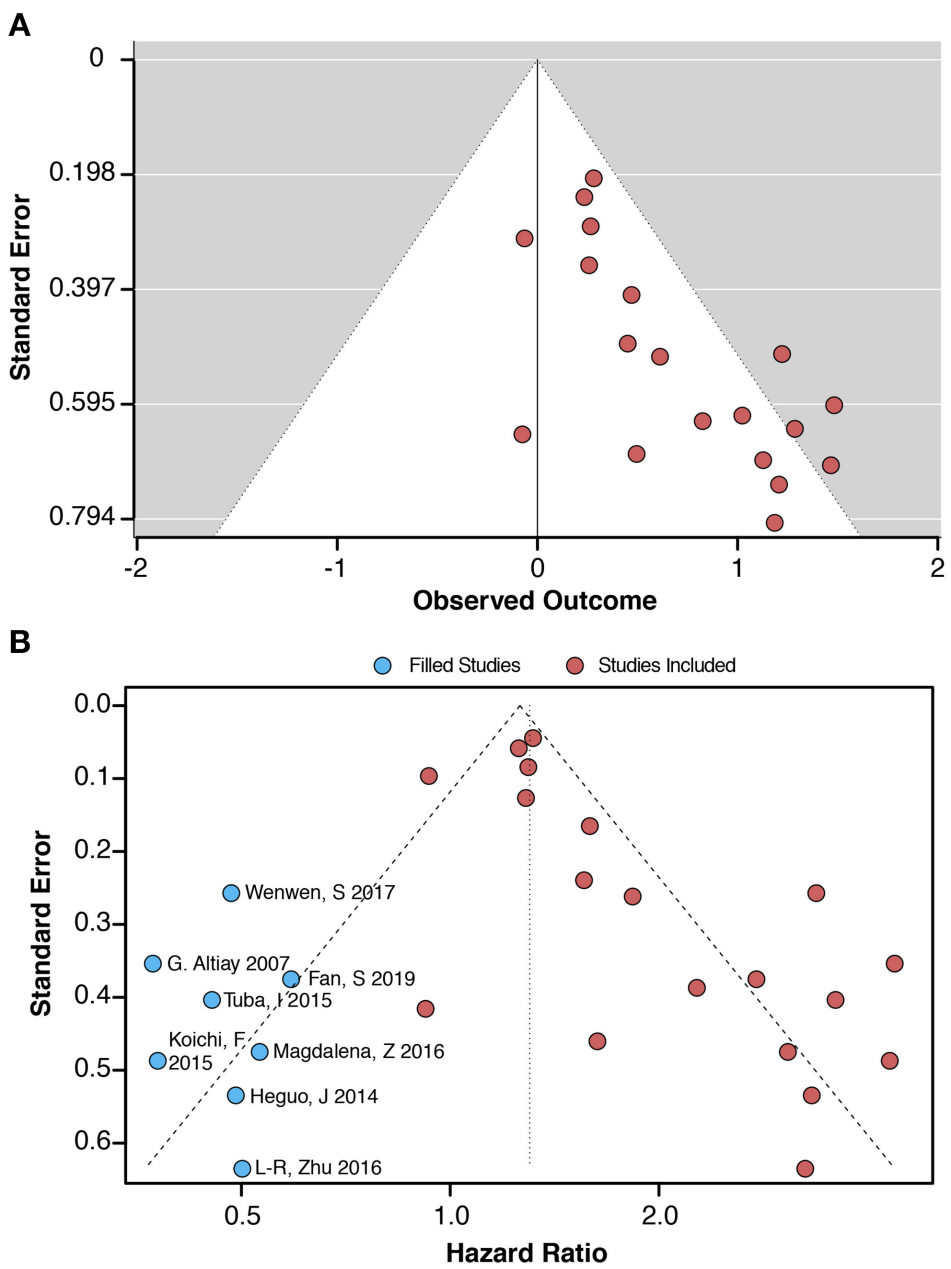
**(A)** Funnel plot for the link between plasma D-dimer levels (highest *vs*. lowest category) and lung cancer-associated mortality. **(B)** Filled funnel plot and meta trim-and-fill model. The blue dots indicate that these included studies require a corresponding study for correction, thus reducing bias.

The data from the funnel plot showed an asymmetry, indicating that publication bias still exists (Figure 3A). Application of the fill and trim model suggested that the inclusion of three new items may eliminate publication bias (Figure 3B). The results of the regression analysis using Begg's (*p* = 0.036) and Egger's (*p* = 0.002) regressions showed the presence of publication bias (Supplementary Figure 2). In addition to this, NOS and standard “risk of bias” tools were used to assess bias (Table 1, Supplementary Figures 3A,B and Supplementary Table 3).

### External Validation

**Figure 4:**
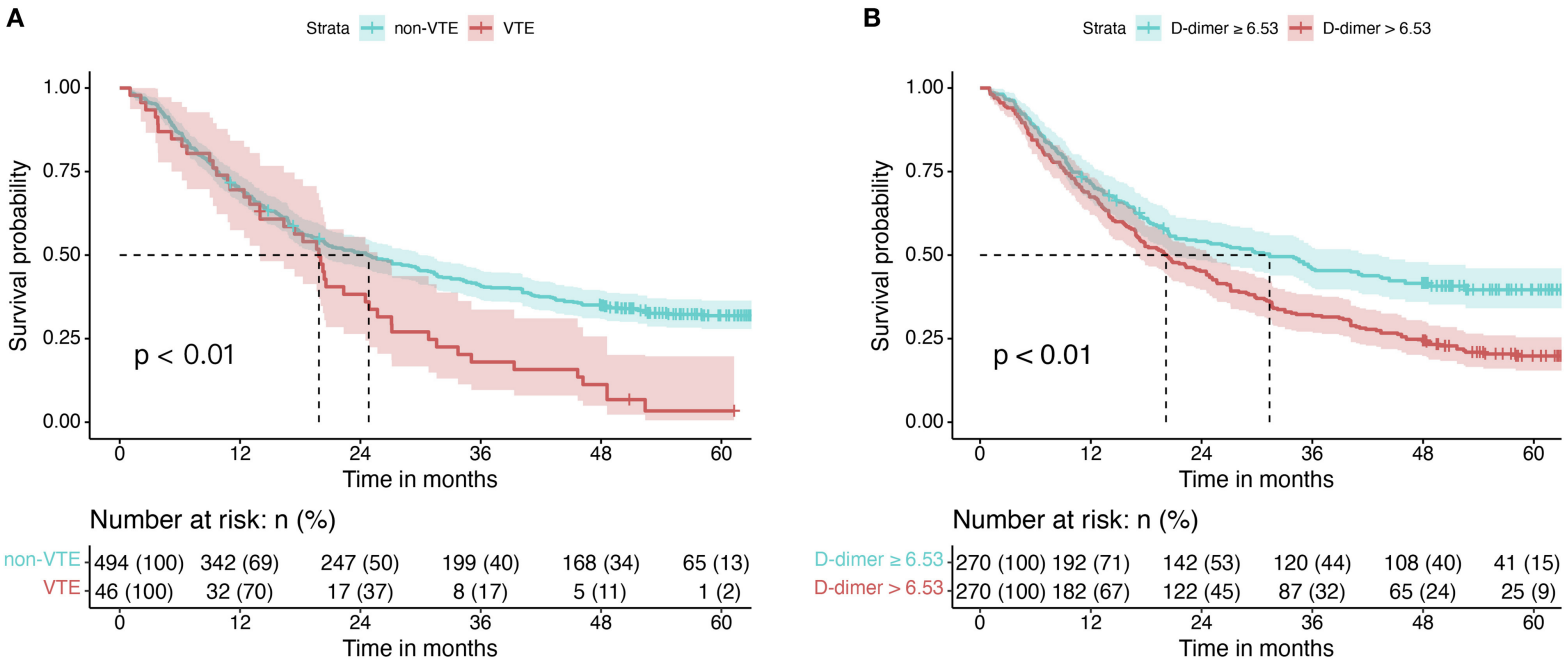
Kaplan-Meier curves for NSCLC patients with different factors. **(A)** Kaplan-Meier curves for OS in patients in the VTE and non-VTE groups. **(B)** Kaplan-Meier curves for OS in different D-dimer levels.

**Figure 5:**
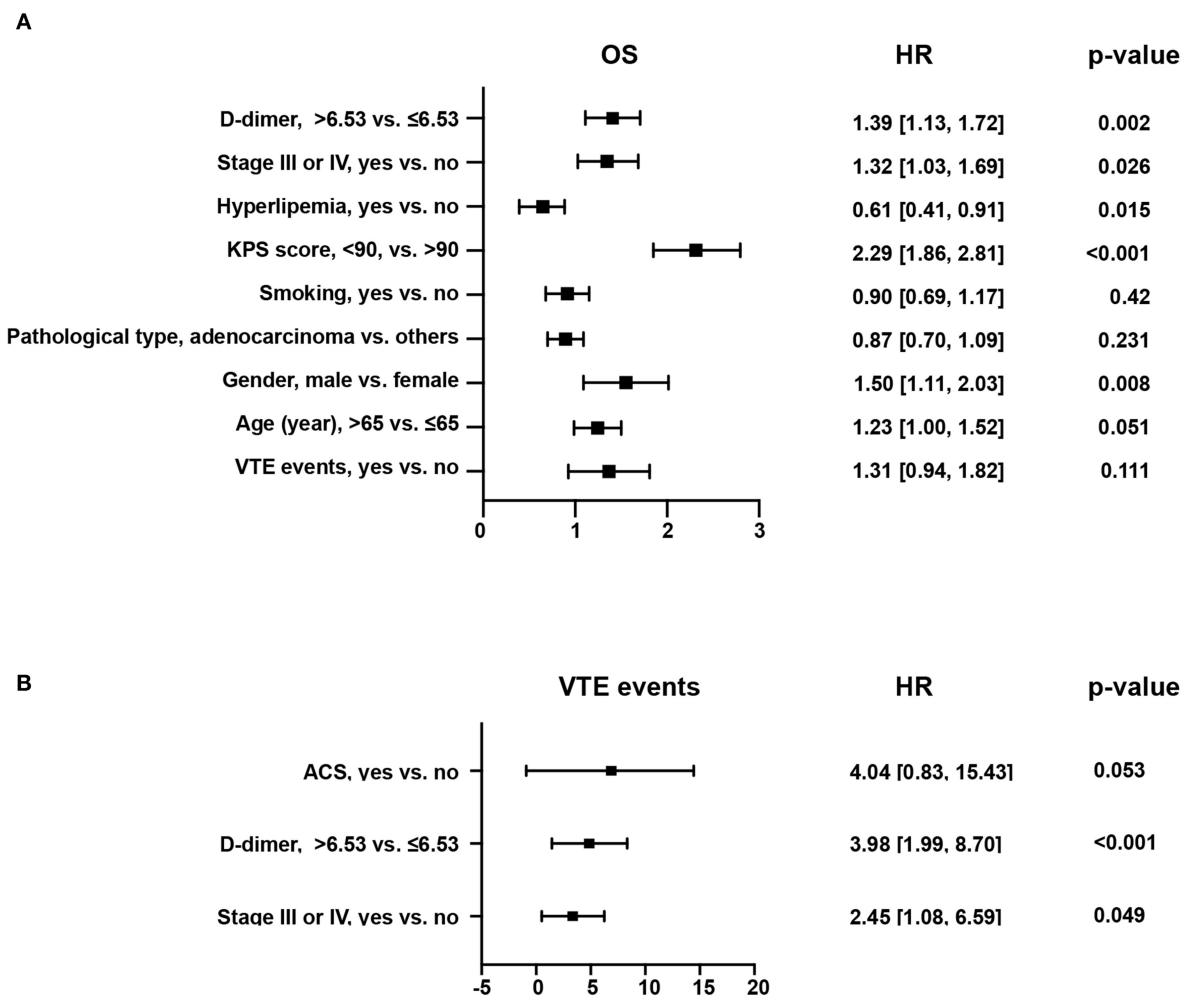
Forest plot for multifactorial analysis of different endpoints in patients with NSCLC. **(A)** Forest plot for multifactor analysis of the risk of overall mortality. **(B)** Forest plot for multifactor analysis of the risk of VTE events.

Five hundred forty patients with lung cancer were included in this project. The median age was 64 (58, 70). Of these, 165 (31.0%) were female and 375 (69.0%) were male. The mean BMI level was 22.64 ± 3.16. 169 (31.0%) cases were squamous carcinomas, and 346 (64.0%) were adenocarcinomas. Smokers accounted for 52.0% of the included population, with 279 cases (Table 3). Kaplan-Meier curves showed that patients with increased levels of VTE events (Figure 4A) or D-dimers (Figure 4B) had a poor prognosis in patients with NSCLC. We chose the median D-dimer of 6.53 as the cut-off value. To explore the effect of D-dimer on long-term prognosis and VTE events in NSCLC patients, we performed a univariate analysis of potential factors. The data showed that stage, D-dimer, hyperlipidemia, type of pathology, KPS score, smoking, gender, VTE events, and age were risk factors for death, whereas ACS, D-dimer, and stage were risk factors for VTE events (Table 4). Based on the statistical results and clinical experience, we included factors with potential predictive value in the multifactorial analysis. The results showed that mortality (HR 1.39, 95% CI: 1.13–1.72, *p* = 0.002; Figure 5A) and VTE events (HR 3.98, 95% CI: 1.99–8.70, *p* = 0.002) in patients with high D-dimer levels (Figure 5B).

**Table 3:** Study Participant Characteristics at Enrollment.

**Variables**	**Total (*n* = 540)**	**Cohort, median (IQR)**		***p*-value**
		**D-dimer ≤6.53 (*n* = 270)**	**D-dimer > 6.53 (*n* = 270)**	
**Baseline data**				
Age, Median (Q1,Q3)	64 (58, 70)	63 (58, 69)	64 (59, 70)	0.144
Gender, n (%)				0.575
female	165 (31)	79 (29)	86 (32)	
male	375 (69)	191 (71)	184 (68)	
BMI, Mean ± SD	22.64 ± 3.16	22.62 ± 3.23	22.66 ± 3.1	0.881
Pathological type, n (%)				0.089
Adenocarcinoma	346 (64)	161 (60)	185 (69)	
Mixed lung cancer	25 (5)	13 (5)	12 (4)	
Squamous carcinoma	169 (31)	96 (36)	73 (27)	
Stage				0.269
I	88 (16)	52 (19)	36 (13)	
II	59 (11)	30 (11)	29 (11)	
III	103 (19)	47 (17)	56 (21)	
IV	290 (54)	141 (52)	149 (55)	
Smoking, n (%)				0.605
No	261 (48)	127 (47)	134 (50)	
Yes	279 (52)	143 (53)	136 (50)	
KPS, Median (Q1,Q3)	90 (80, 90)	90 (80, 90)	90 (80, 90)	0.133
D-dimer, Median (Q1,Q3) (U/L)	6.53 (3.94, 8.48)	3.93 (2.85, 5.42)	8.49 (7.4, 10.34)	<0.001
**Complications**				
Hypertension, n (%)				0.037
No	366 (62)	49 (53)	47 (76)	
Yes	224 (38)	44 (47)	15 (24)	
Diabetes, n (%)				0.323
No	533 (90)	82 (88)	57 (92)	
Yes	57 (10)	11 (12)	5 (8)	
Hyperlipidemia, n (%)				0.024
No	538 (91)	88 (95)	57 (92)	
Yes	52 (9)	5 (5)	5 (8)	
Heart.failure, n (%)				0.111
No	579 (98)	89 (96)	61 (98)	
Yes	11 (2)	4 (4)	1 (2)	
ACS, n (%)				0.813
No	575 (97)	90 (97)	60 (97)	
Yes	15 (3)	3 (3)	2 (3)	
**Outcomes**				
Status, n (%)				<0.001
alive	174 (29)	43 (46)	23 (37)	
dead	416 (71)	50 (54)	39 (63)	
Overall survival time, Median (Q1,Q3)	24.17 (9.9, 50.99)	38.9 (12.87, 59.8)	18.05 (7.53, 50.99)	0.012
VTE events				<0.001
	494 (91)	260 (96)	234 (87)	
	46 (9)	10 (4)	36 (13)	

*IQR, interquartile range; BMI, Body Mass Index; KPS, Karnofsky Performance Status; AQI, air quality index; ACS, Acute Coronary Syndromes; VTE, venous thromboembolism*.

**Table 4:** Univariate regression analysis on NSCLC patients for different endpoints.

**Variants**	**OS**		**VTE events**	
	**HR**	***p*-value**	**OR**	***p*-value**
VTE events, yes vs. no	1.72 [1.25, 2.36]	0.001	-	-
Age (year), >65 vs. ≤ 65	1.39 [1.13, 1.70]	0.002	0.79 [0.42, 1.47]	0.466
Gender, male vs. female	1.45 [1.15, 1.82]	0.001	1.27 [0.66, 2.62]	0.492
Pathological type, adenocarcinoma vs. others	0.80 [0.65, 0.99]	0.036	1.06 [0.57, 2.03]	0.866
Stage III or IV, yes vs. no	1.32 [1.04, 1.68]	0.024	2.66 [1.19, 7.12]	0.029
Smoking, yes vs. no	1.23 [1.00, 1.51]	0.045	1.24 [0.68, 2.30]	0.491
KPS score, <90, vs. >90	2.34 [1.91, 2.88]	<0.001	1.45 [0.79, 2.67]	0.228
D-dimer, >6.53 vs. ≤ 6.53	1.51 [1.23, 1.85]	<0.001	4.00 [2.01, 8.68]	<0.001
Hypertension, yes vs. no	1.04 [0.84, 1.27]	0.743	1.17 [0.62, 2.14]	0.626
Diabetes, yes vs. no	1.10 [0.78, 1.54]	0.591	-	-
Hyperlipidemia, yes vs. no	0.66 [0.45, 0.98]	0.037	1.08 [0.31, 2.85]	0.887
Heart failure, yes vs. no	1.25 [0.59, 2.63]	0.563	1.20 [0.06, 6.59]	0.866
ACS, yes vs. no	1.45 [0.77, 2.72]	0.245	3.06 [0.67, 10.26]	0.095
BMI, >24 vs. ≤ 24	0.94 [0.75, 1.17]	0.554	0.81 [0.39, 1.57]	0.545

*IQR, interquartile range; BMI, Body Mass Index; KPS, Karnofsky Performance Status; AQI, air quality index; ACS, Acute Coronary Syndromes*.

## Discussion

In this project, by pooling 19 cohort studies on D-dimer and lung cancer involving a total of 5819 patients, we found that high circulating plasma D-dimer levels were associated with the risk of death in lung cancer patients. And external validation not only demonstrated the above correlation and found that its elevated levels were equally related to the occurrence of long-term VTE events. During the analysis, we detected significant heterogeneity, and further subgroup analysis revealed that disease status might be the primary source of heterogeneity (p <0.001). Characteristics such as days of follow-up, country, public year, design, quality score, and population were not strongly correlated for the association of plasma D-dimer with the endpoint event of death in lung cancer patients. The above data suggest that lung cancer patients with higher plasma D-dimer have a poor long-term prognosis.

D-dimer is regarded as a biomarker of intravascular fibrin degradation and formation (33). It is also a biomarker of the activation of coagulation (34). Previous studies showed that the production of D-dimer might be stimulated by hypercoagulability, such as cancer, chronic inflammation, aging, as well as atrial fibrillation (35).

D-dimers are produced during the generation of fibrin monomers in fibrinogen, which consists of two peripheral D-structural domains and E-structural domain linkage centers. The fibrin network is formed. After conversion, the D-domain is cross-linked by factor XIII, strengthening the fibrin network. Thrombin bound to fibrin is degraded from the fibrin network to soluble fragments. Thereby D dimers are produced. Thus, D-dimer in peripheral blood reflects the degree of fibrinolysis and coagulation and is elevated during thrombosis (34).

The process of coagulation is the conversion of blood from a liquid to a gel, which then forms a blood clot. Recent investigations suggested that patients with lung cancer showed disorders in activating both fibrinolysis and coagulation. Over the past few years, many studies explored the mechanism of hypercoagulability and thrombogenesis in tumor status. Cancer cells cause coagulation relying on their apparent procoagulant activity to activate the coagulation system and deposit fibrin (36). Meanwhile, cancer cells could induce hypercoagulable status by secreting procoagulant factors, circulating microparticles, enhancing endothelial function, and altering platelet activity (37).

Moreover, some chemotherapeutics, especially platinum compounds, have been reported to increase the risk of thrombosis (7). Recent investigations have found that regulating hemostatic protein expression depends on oncogenes. The tumor-derived tissue factor-positive microparticles play a critical role in thrombosis (38). There is evidence that membrane vesicles of tumor cells can activate the coagulation system. In contrast, microvesicles (MV) released from the membranes of apoptotic cells can stimulate coagulation via negatively charged membrane phospholipids or tissue factors (TF). Several types of cancer cells can release MV-derived TF *in vitro*, but the function of cancer cells *in vivo* still needs to be further explored (39, 40).

The incidence of thrombosis in patients is increased. In addition, the initiation of the hemostatic system affects every stage of the tumor (14). Tumors can activate coagulation factors and induce a procoagulant state, promoting tumor invasion, angiogenesis, growth, and metastasis (41, 42). Deposition of fibrin in lung cancer tissues may promote the generation of new blood vessels and the proliferation of cancer cells. These deposits could protect the tumor cells against chemotherapy drugs and immune elimination (43, 44). Besides, tumor cells may cause intravascular clotting when entering the blood and promoting metastasis (45). Osamu T et al. reported that circulating biomarkers of the fibrinolysis system play a significant role in response to chemotherapy, tumor burden, and clinical progression in lung cancer (46). Previous studies have shown that a history of hyperlipidemia and serum D-dimer can together predict the probability of stroke events in oncology patients, and this may also be promising for lung cancer patients (47).

VTE is a recognized complication of cancer, and the incidence of VTE in cancer patients is more than four times that of normal controls. d-dimer has been used as a predictor of VTE in lung cancer patients, so we hypothesized that it could also be used as a biomarker for lung cancer (48). Cancer patients have a high risk of developing VTE, which leads to severe consequences that cover the need for long-term anticoagulant therapy (49). The risk of VTE is further increased by chemotherapeutic or immunomodulatory drugs (50). Besides venous thrombosis, arterial thrombosis is also a common complication of lung cancer and predicts a poor prognosis in common cancers (7, 51). Also, DIC further complicates the spectrum of hemostatic complications in malignancy, which is fatal (41).

However, among the papers we analyzed in this meta-analysis, two studies showed an increased D-dimer predicts a good prognosis (17, 18). Moreover, these two studies were also a source of heterogeneity. The study conducted by Zhang et al. (17) collected the plasma before chemotherapy for D-dimer detection. It may be responsible for the negative result of the link between D-dimer and the prognosis. In another investigation by Ge et al. (18), they only compared the patients in the B0 stage, so their result also displayed a negative link between D-dimer and mortality of patients with lung cancer.

## Limitations and Strength

We found the prognostic, predictive value of D-dimer in different pathological types of lung cancer by meta-analysis. Few previous studies have meta-analyses showing the correlation between D-dimer and lung cancer mortality endpoints. Our study included only prospective cohorts and followed MOOSE and PRISMA guidelines. Sources of heterogeneity were analyzed using different pathways, including subgroup analysis, meta-regression, and padding and trimming methods were used to explore the publication bias.

However, our study has limitations. First, not all study items were included because data could not be extracted for some studies, which may raise the risk of bias. Most of the studies we included were of NSCLC patients and the number of SCLC patients was small, so there will be some bias. Future prospective studies are needed for relevant validation. In addition, there was considerable bias in the underlying clinical characteristics of each program, including race, sex, and age in the individual studies. Therefore, whether increased D-dimer is associated with the occurrence of lung cancer mortality endpoints requires a large sample size of clinical trials to verify.

## Conclusion

Elevated circulating D-dimer levels correlate with the endpoint of death and VTE development in lung cancer patients. However, large sample size clinical trials are still needed in the future to validate this argument.

## Data Availability Statement

The raw data supporting the conclusions of this article will be made available by the authors, without undue reservation.

## Ethics Statement

Written informed consent was obtained from the individual(s) for the publication of any potentially identifiable images or data included in this article.

## Author Contributions

JL and SY designed and performed research studies, analyzed the data, and wrote the manuscript. XZ, MX, CZ, LG, CY, and XW performed research studies and analyzed data. SC, DZ, and JJ contributed to the research design, data analysis, manuscript writing, and study supervision. All authors contributed to the article and approved the submitted version.

## Funding

This study was supported by the Social Development Project of Jiangsu Province, China in 2016 (no. BE2016672) and the Project of Hygiene and Health Committee of Jiangsu Province, China (no. H2019028).

## Conflict of Interest

The authors declare that the research was conducted in the absence of any commercial or financial relationships that could be construed as a potential conflict of interest.

## Publisher's Note

All claims expressed in this article are solely those of the authors and do not necessarily represent those of their affiliated organizations, or those of the publisher, the editors and the reviewers. Any product that may be evaluated in this article, or claim that may be made by its manufacturer, is not guaranteed or endorsed by the publisher.

## Abbreviations

NSCLC: non-small-cell lung cancer
NALC: non-advanced lung cancer
ALC: advanced lung cancer
PCS: prospective cohort study
LC: lung cancer
IA: immunoturbidimetric assay
ELISA: enzyme-linked immunosorbent assay
SCLC: small cell lung cancer
RS: retrospective study
RCT, randomized controlled trial, HR: hazard ratio.
