# Supplementary material for: Circulating D-Dimers Increase the Risk of Mortality and Venous Thromboembolism in Patients With Lung Cancer: A Systematic Analysis Combined With External Validation
Source: Front Med (Lausanne). 2022 Mar 2;9:853941. doi: 10.3389/fmed.2022.853941 (PMC8924589; doi:10.3389/fmed.2022.853941)
Supplement: Supplementary file 1 [file Data_Sheet_1.PDF]

Supplement Materials

Figure Legends

Fig. S1. Sensitivity analysis for plasma D-dimer level and lung cancer associated mortality.

Fig. S2. Begg's test plot (**A**) and Egger linear regression test (**B**) with 95% CI for the relationship between plasma D-dimer level and lung cancer associated mortality.

Fig. S3A. Risk of bias graph: review author's judgements about each risk of bias item percentages across all included studies.

Fig. S3B. Risk of bias summary: review author's judgements about each risk of bias item percentages across all included studies.

Table S1. Quality of Evidence evaluated by GRADE system

Author	Year	Design	Downgrade quality of evidence					Upgrade quality of evidence			Quality of Evidence
			Risk of Bias	Inconsistencey	Indirectness	Imprecision	Publication Bias	Large effect	PCWCE	Dose-response Gradi	
Fan, S	2019	RS	serious(-1)	serious(-1)	no	serious(-1)	serious(-1)	no	no	no	Low ⊕⊕□□
Minglei, Y	2019	RCT	no	no	serious(-1)	no	undetected	large (+1)	no	no	High ⊕⊕⊕⊕
Hou, C	2019	PCS	no	serious(-1)	no	no	undetected	no	no	no	Moderate ⊕⊕⊕□
Huangliang, L	2019	RS	serious(-1)	no	serious(-1)	no	serious(-1)	no	serious(-1)	no	Low ⊕⊕□□
Edyta I. W	2018	PCS	no	serious(-1)	no	no	undetected	no	no	no	Moderate ⊕⊕⊕□
Cuicui, Z	2018	RS	serious(-1)	no	serious(-1)	no	serious(-1)	no	serious(-1)	no	Low ⊕⊕□□
Wenwen, S	2017	PCS	no	serious(-1)	no	no	undetected	no	no	no	Moderate ⊕⊕⊕□
Kaoru, S	2017	PCS	no	no	serious(-1)	no	undetected	no	no	no	Moderate ⊕⊕⊕□
L-R, Zhu	2016	PCS	no	no	no	no	undetected	large (+1)	no	no	High ⊕⊕⊕⊕
Magdalena, Z	2016	PCS	serious(-1)	no	no	no	undetected	no	no	no	Moderate ⊕⊕⊕□
Koichi, F	2015	PCS	no	no	serious(-1)	no	undetected	no	no	no	Moderate ⊕⊕⊕□
Yuezhen, W	2015	RS	serious(-1)	serious(-1)	no	serious(-1)	undetected	no	no	no	Low ⊕⊕□□
Tuba, I	2015	PCS	no	serious(-1)	no	no	undetected	no	no	no	Moderate ⊕⊕⊕□
L-P, Ge	2014	PCS	no	no	serious(-1)	no	undetected	no	no	no	Moderate ⊕⊕⊕□
Heguo, J	2014	PCS	no	serious(-1)	no	no	undetected	no	no	no	High ⊕⊕⊕⊕
P-P, Zhang	2013	PCS	no	no	no	no	undetected	no	no	no	Moderate ⊕⊕⊕□
Cihan, A	2012	PCS	no	no	serious(-1)	no	undetected	large (+1)	no	no	Moderate ⊕⊕⊕□
Katsuhiro, M	2011	PCS	no	serious(-1)	no	no	undetected	no	no	no	Moderate ⊕⊕⊕□
G. Altiay	2007	PCS	serious(-1)	no	no	no	undetected	no	no	no	Moderate ⊕⊕⊕□

Table S2. Overview of multivariable relationship of D-dimer with lung cancer

Author	Year	HR [95% CI]	Adjusted Covariate
Fan, S	2019	2.73 [1.32-5.63]	KPS score, N-Stage, TNM-Stage, Treatment, NSE
Minglei, Y	2019	1.28 [1.1-1.5]	Age, Visceral metastases, Number of metastases in the vertebral body, Frankel score, EGFR mutation
Hou, C	2019	1.61 [0.66-3.93]	Gender, anatomic location, LVI, VPI, TNM stage, and tumor size.
Huangliang, L	2019	1.27 [1-1.61]	Smoking, Lesion type, Resection type T stage, Lymph node metastasis Fibrinogen level, NLR, PLR, LMR
Edyta I. W	2018	1.81 [1.09-2.98]	None
Cuicui, Z	2018	0.92 [0.86-1.23]	None
Wenwen, S	2017	3.33 [2.03-5.45]	None
Kaoru, S	2017	2.24 [1.05-4.69]	Age, Serum CEA, SUVmax of primary tumor, Pleural invasion, Pathological stage Stage
L-R, Zhu	2016	3.21 [0.96-11.34]	Age, gender, ECOG-PS, Tumor Stage, Response to chemotherapy, Fibrinogen, NSE, CEA, LDH
Magdalena, Z	2016	3.03 [1.22-7.69]	Age, Rash, Dominant histological diagnosis, Deletion in exon 19, Substitution in exon 21, Clinical stage, Karnofsky performance, Gender, Cardiovascular diseases, Prophylaxis with LWMH, Smoking, Previous thoracic surgery, Previous radiotherapy, Previous chemotherapy, Pre-treatment level of D-dimer
Koichi, F	2015	4.25 [1.65-10.91]	Age, Sex, Smoking history, p-stage, Histology, Surgical procedure, CEA
Yuezhen, W	2015	1.24 [1.12-1.38]	Sex, Age, smoking history, histology, TNM stage, PS
Tuba, I	2015	3.55 [1.63-7.77]	Hemoglobin pretreatment, APTT, LDH
L-P, Ge	2014	0.91 [0.35-1.75]	Tumor stage, Number of metastatic sites, Treatment response, CEA, Cyfra 21-1
Heguo, J	2014	3.28 [1.12-8.92]	Lymph node status, Pathologic stage, Plasma fibrinogen, Serum CEA,Serum Cyfra 21-1
P-P, Zhang	2013	1.54 [1.11-2.78]	Age, Sex, Histology, Tumor size, TNM stage, VTE, Surgery,
Cihan, A	2012	1.30 [1.20-1.40]	Gender, Age, Different tumor groups, Venous thromboembolism
Katsuhiro, M	2011	1.57 [1.17-2.19]	None
G. Altiay	2007	4.32 [2.18-8.55]	Tumour stage, Karnofsky performance, status

Abbreviation: KPS, Karnofsky Performance Status; NSE, neuron Speoific enolase; LVI, lymphovascular invasion; VPI, visceral pleural invasion; NLR, neutrophil-lymphocyte ratio; PLR, platelet-lymphocyte ratio; LMR, lymphocyte-monocyte ratio; HR, hazard ratio; CEA, carcinoembryonic antigen; LDH, lactate dehydrogenase; ECOG, Eastern Cooperative Oncology Group; PS, performance status; LWMH, low molecular weight heparin; APTT, activated partial thromboplastin time.

Figure S1

Study	HR	95%-CI
Omitting Fan, S 2019	1.30	[1.24; 1.37]
Omitting Minglei, Y 2019	1.31	[1.24; 1.38]
Omitting Hou, C 2019	1.31	[1.24; 1.37]
Omitting Huangliang, L 2019	1.31	[1.24; 1.38]
Omitting Edyta I. W 2018	1.30	[1.24; 1.37]
Omitting Cuicui, Z 2018	1.35	[1.28; 1.42]
Omitting Wenwen, S 2017	1.29	[1.23; 1.36]
Omitting Kaoru, S 2017	1.30	[1.24; 1.37]
Omitting L-R, Zhu 2016	1.30	[1.24; 1.37]
Omitting Magdalena, Z 2016	1.30	[1.24; 1.37]
Omitting Koichi, F 2015	1.30	[1.24; 1.37]
Omitting Yuezhen, W 2015	1.33	[1.25; 1.41]
Omitting Tuba, I 2015	1.30	[1.24; 1.37]
Omitting L-P, Ge 2014	1.31	[1.24; 1.38]
Omitting Heguo, J 2014	1.30	[1.24; 1.37]
Omitting P-P, Zhang 2013	1.30	[1.24; 1.37]
Omitting Cihan, A 2012	1.31	[1.23; 1.40]
Omitting Katsuhiro, M 2011	1.30	[1.23; 1.37]
Omitting G. Altiay 2007	1.30	[1.23; 1.37]
**Fixed effect model**	**1.31**	**[1.24; 1.37]**

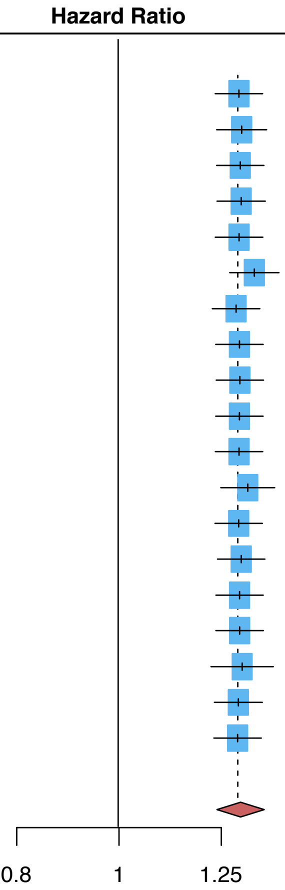

Figure S2A

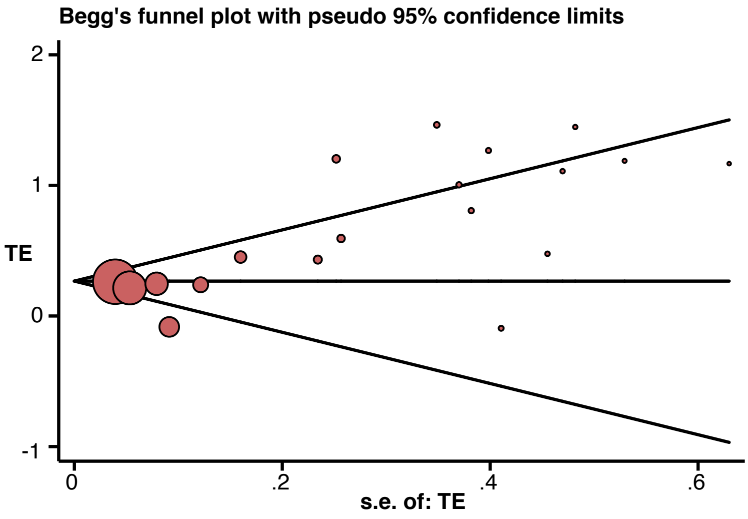

Figure S2B

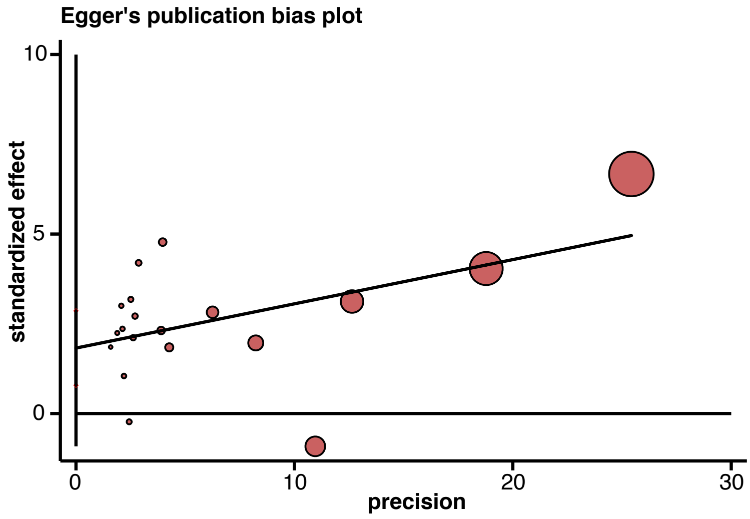

Figure S3A

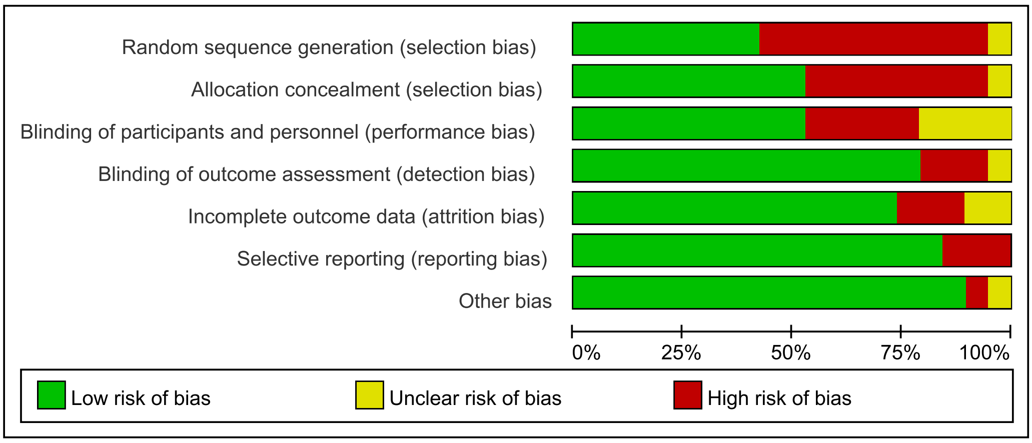

Figure S3B

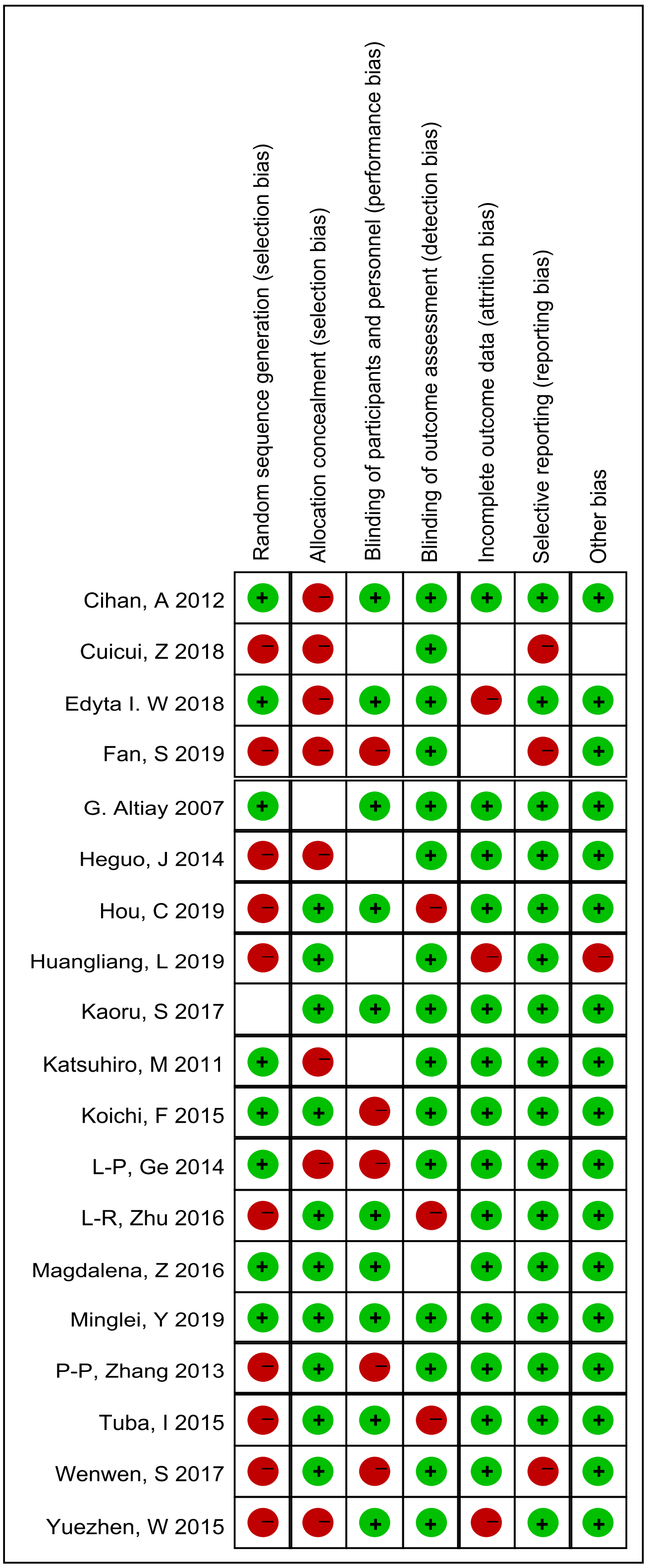
Random sequence generation (selection bias)
Allocation concealment (selection bias)
Blinding of participants and personnel (performance bias)
Blinding of outcome assessment (detection bias)
Incomplete outcome data (attrition bias)
Selective reporting (reporting bias)
Other bias
Cihan, A 2012
Cuicui, Z 2018
Edyta I. W 2018
Fan, S 2019
G. Altiay 2007
Heguo, J 2014
Hou, C 2019
Huangliang, L 2019
Kaoru, S 2017
Katsuhiro, M 2011
Koichi, F 2015
L-P, Ge 2014
L-R, Zhu 2016
Magdalena, Z 2016
Minglei, Y 2019
P-P, Zhang 2013
Tuba, I 2015
Wenwen, S 2017
Yuezhen, W 2015
